# Syringocystadenoma papilliferum in the scalp, with a linear presentation^☆^

**DOI:** 10.1016/j.abd.2021.11.011

**Published:** 2023-01-06

**Authors:** Marina Monaco, Virginia Mariana González, Felix Alberto Vigovich, Margarita Larralde

**Affiliations:** a Department of Dermatology, Hospital Alemán, Buenos Aires, Argentina; b Department of Pathology, Hospital Alemán, Buenos Aires, Argentina

Dear Editor,

Syringocystadenoma Papilliferum (SCAP) is an uncommon benign adnexal tumor that originates from pluripotent cells with either apocrine or eccrine sweat gland differentiation. Around 50% of cases are present at birth while 15%‒30% of them can develop during puberty.^1^ SCAP can be seen as a *de novo* tumor, without any preexisting lesion or it can coexist with both benign or malignant tumors.^2^ The SCAP is usually located in the head and neck region. When located on the scalp, it is frequently associated with the nevus sebaceous of Jadassohn.^3^

There are varied clinical presentations. It can appear as a plaque-type, as a solitary papule, or as several papules with a linear arrangement as in our case.^4^, ^5^, ^6^

The linear presentation of SCAP is extremely rare accounting for less than 20 cases reported in the literature.^6^ The treatment is surgical excision. In patients with unfavorable anatomic sites for surgery, a CO_2_ laser may be effective.^7^

We report a case of SCAP with an atypical presentation given its size, linear arrangement, and in absence of a preexisting nevus sebaceous.

The aim of our case report consists of the communication of the dermatoscopic features in order to contribute this data to the scientific community due to the lack of information in the literature.

A 31-year-old man was evaluated for a congenital scalp lesion. The patient noticed that the tumor had increased in size during the past 10 years. A serous discharge was detected and occasionally bleeding at minimal trauma.

The physical examination revealed a pinkish-erythematous tumor with linear distribution in the occipital region. It was characterized by multiple grouped papules measuring 3.5 × 1.5 cm. The surface of the papules presented central umbilication and crusting. Their base was not indurated and there wasn’t evidence of local lymphadenopathy (Fig. 1).

**Figure 1:**
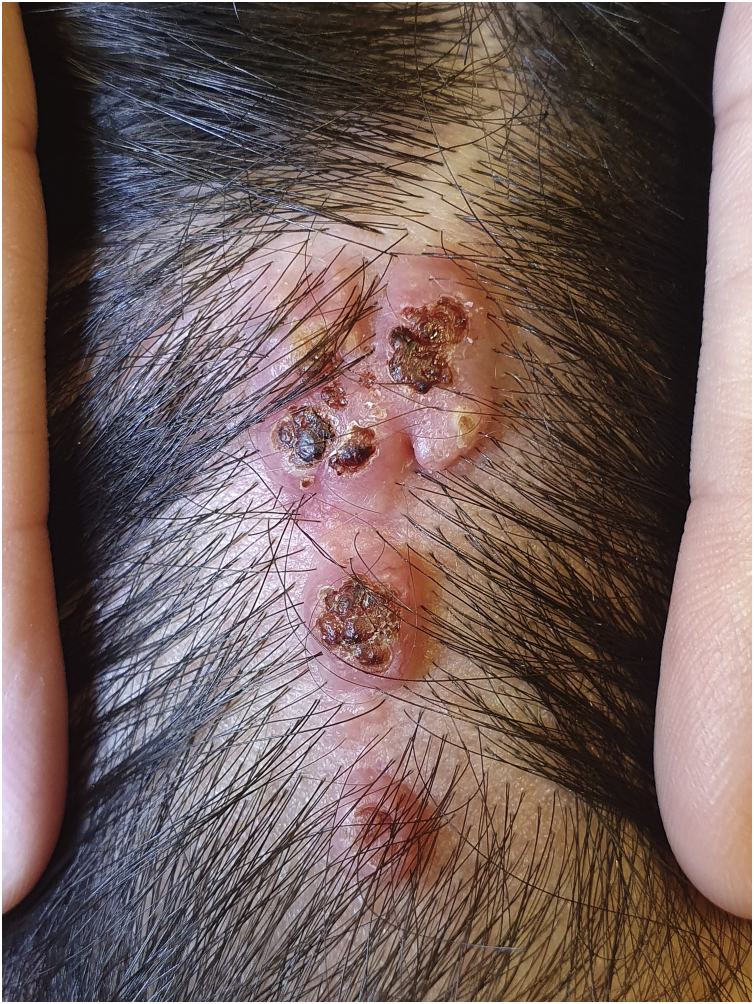
Linear SCAP of the scalp, conformed by multiple grouped papules with central umbilication and crusted surface.

The dermoscopic examination showed red papillomatous projections with central ulceration in an erythematous background. Some presented white circles and crusted erosive centers. Polymorphic vessels were seen within the periphery of the lesion (Fig. 2).

**Figure 2:**
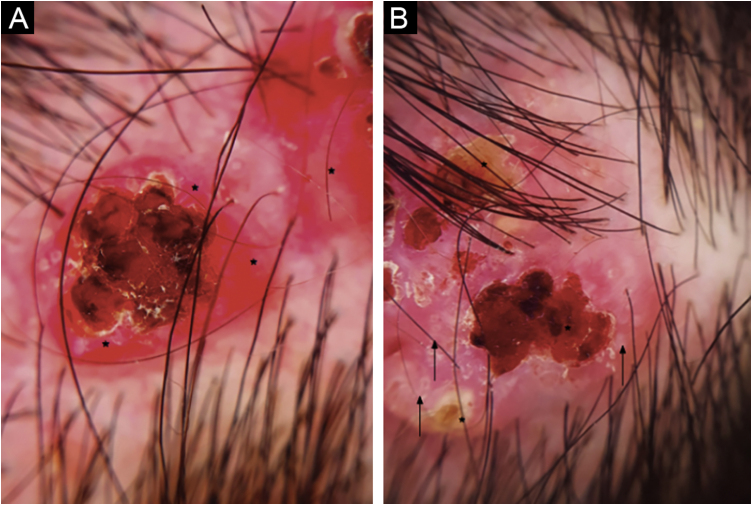
(A) Red papillomatous projection with central ulceration in an erythematous background and polymorphic vessels in the periphery of the lesion (asterisk) (B) Another lesion with multiple ulcerations, whitish-yellow crusts (asterisk), and small white circles (arrow) on the periphery.

The histopathological study revealed cystic invaginations extending downwards from the epidermis with numerous papillary projections protruding into the lumen, (Fig. 3A), lined by two layers of glandular epithelium. The cells of the inner layer were columnar with oval nuclei and eosinophilic cytoplasm, while the outer layer cells were smaller, cuboidal, and had oval nuclei with scanty cytoplasm (Fig. 3B). The lesion was successfully treated with surgical excision without recurrence.

**Figure 3:**
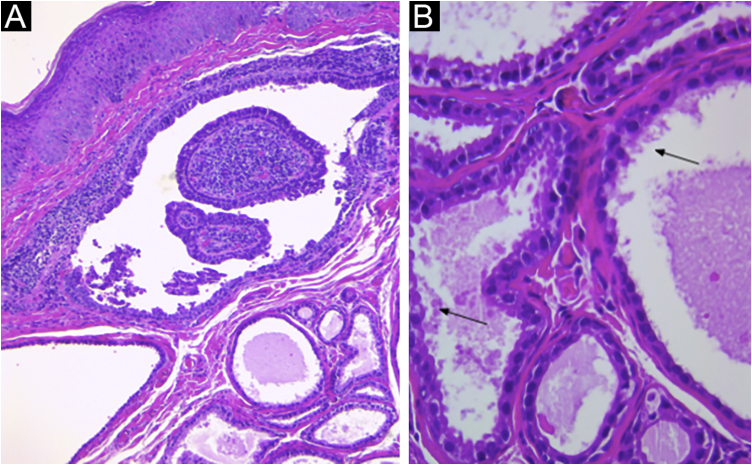
(A) Irregular papillary projections protruding into the lumen with plasmocytes in the core (Hematoxylin & eosin, 100×). (B) Cystic spaces are lined by two layers of glandular epithelium. Cells of the inner layer are columnar with oval nuclei, while in the outer layer, the cells are smaller, cuboidal, and have oval nuclei with scatty cytoplasm. Decapitation on the luminal surface can be noticed (↙) (Hematoxylin & eosin, 400×).

SCAP is an uncommon skin adnexal tumor of apocrine or eccrine sweat gland origin. It usually manifests as a solitary lesion.^4^ It is observed at birth in 50% of cases and in 15%‒30% of them it develops during puberty.^6^, ^3^ The most reported localization is the head and neck area, up to 75% of the cases and, 40% of them develop in association with a pre-existing nevus sebaceous. There are multiple other anatomical sites of involvement.^4^, ^7^, ^8^

The lesions are usually skin colored to pink, hairless, firm plaques, and solitary or grouped nodules.^4^ These may present with smooth flat or dome-shaped surfaces. The larger lesions may ulcerate. They tend to increase in size at puberty and sometimes multiply in number as well as becoming more verrucous and papillomatous.^7^ Some can show central umbilication, small fistulae with serous fluid discharge, and bleeding secondary to minimal trauma.^4^

Dermatoscopic findings include red exophytic papillary structures that can present with central umbilication and ulceration that can acquire ragged or polylobulate edges. These are not usually seen in other tumors such as Basal Cell Carcinoma (BCC). Some can present white circles, central crust, yellowish scales, and pinkish-white globular structures. There can be seen hairpin vessels, polymorphous and coma vessels. In contrast in nodular BCC, the expected vessels are usually branched linear vessels. In addition, the presence of scales also excludes BCC. SACP in association with nevus sebaceous, had been described also irregular dotted, glomerular, and linear vessels with a pinkish-white circumference and peripheral hairpin-like vessels.^6^ Given the rarity of the pathology, there are small series of cases that don’t establish sensitivity and specificity of the dermoscopic structures. Our case presents dermoscopic similarities to the case described by Chauhan et al.

The diagnosis requires histopathological confirmation. Among the differential diagnosis are included hidradenoma papilliferum and papillary eccrine adenoma.^4^

Despite SCAP is most frequently associated to nevus sebaceous, it might develop on its surface BCC, sebaceous epithelioma, apocrine hydrocystadenoma, trichoepithelioma, eccrine spiroadenoma, and tubular apocrine adenoma.^9^, ^10^

BCC development has been reported in up to 10% of cases. Squamous Cell Carcinoma (SCC) can also be associated, but less frequently. Verrucous carcinoma and ductal carcinoma had also been reported.^5^, ^2^ If the lesion evolves with ulceration or a rapid enlargement it can be indicative of a malignant transformation, such as in the case of a syringocystadenocarcinoma papilliferum.^5^ Cases of linear SCAP type turning into malignancy are yet to be reported.

## Financial support

None declared.

## Authors’ contributions

Marina Monaco: Approval of the final version of the manuscript; critical literature review; data collection, analysis and interpretation; effective participation in research orientation; manuscript critical review; preparation and writing of the manuscript; study conception and planning.

Virginia Mariana Gonzalez: Approval of the final version of the manuscript; critical literature review; data collection, analysis and interpretation; effective participation in research orientation; intellectual participation in propaedeutic and/or therapeutic: management of studied cases; manuscript critical review; preparation and writing of the manuscript; statistical analysis; study conception and planning.

Felix Alberto Vigovich: Approval of the final version of the manuscript; critical literature review; manuscript critical review; preparation and writing of the manuscript.

Margarita Larralde: Approval of the final version of the manuscript; critical literature review; data collection, analysis and interpretation; intellectual participation in propaedeutic and/or therapeutic: management of studied cases; manuscript critical review; preparation and writing of the manuscript.

## Conflicts of interest

None declared.
